# Antimicrobial activity of bovine NK-lysin-derived peptides on *Mycoplasma bovis*

**DOI:** 10.1371/journal.pone.0197677

**Published:** 2018-05-17

**Authors:** Rohana P. Dassanayake, Shollie M. Falkenberg, Karen B. Register, Daniel Samorodnitsky, Eric M. Nicholson, Timothy A. Reinhardt

**Affiliations:** 1 Ruminant Diseases and Immunology Research Unit, National Animal Disease Center, Agricultural Research Service, United Sates Department of Agriculture, Ames, Iowa, United States of America; 2 Virus and Prion Research Unit, National Animal Disease Center, Agricultural Research Service, United States Department of Agriculture, Ames, Iowa, United States of America; The University of Melbourne, AUSTRALIA

## Abstract

Antimicrobial peptides (AMPs) are a diverse group of molecules which play an important role in the innate immune response. Bovine NK-lysins, a type of AMP, have been predominantly found in the granules of cytotoxic T-lymphocytes and NK-cells. Bovine NK-lysin-derived peptides demonstrate antimicrobial activity against various bacterial pathogens, including several involved in bovine respiratory disease complex (BRDC) in cattle; however, such studies are yet to be performed with one important contributor to the BRDC, *Mycoplasma bovis*. Therefore, the goal of this study was to assess the antimicrobial activity of bovine NK-lysin-derived peptides on *M*. *bovis*. Thirty-mer synthetic peptides corresponding to the functional region helices 2 and 3 of bovine NK-lysins NK1, NK2A, NK2B, and NK2C were evaluated for killing activity on *M*. *bovis* isolates. Among four peptides, NK2A and NK2C showed the highest antimicrobial activity against the *M*. *bovis* isolates tested. All four NK-lysin peptides induced rapid plasma membrane depolarization in *M*. *bovis* at two concentrations tested. However, based on propidium iodide uptake, only NK2A and NK2C appeared capable of causing structural damage to *M*. *bovis* plasma membrane. Confocal microscopy, flow cytometry, and transmission electron microscopy further suggested NK-lysin-induced damage to the plasma membrane. Taken together, the findings in this study suggest that plasma membrane depolarization alone was insufficient to induce lethality, but disruption/permeabilization of the *M*. *bovis* plasma membrane was the cause of lethality.

## Introduction

Antimicrobial peptides (AMPs), which consist of a varying number of amino acids, are a diverse group of molecules and serve as a first line host innate defense mechanism. AMPs have been identified in many species such as bacteria, insects, birds, fish, amphibians, plants, and mammals [1–4]. AMPs are highly effective against a wide variety of pathogens including bacteria, fungi, parasites and viruses [5–8]. As of 2013, over 5,500 natural (3904) and synthetic (1,643) peptides have been identified [9]. Based on the amino acid composition and structure, AMPs can be divided into five subgroups [10]. Although the mode of action for most AMPs is poorly understood, the majority of AMPs are believed to interact with the lipid bilayer leading to membrane pore formation resulting in microbial cell lysis [11]. However, there are a few AMPs, such as buforin and nisin which do not target bacterial cell membranes for killing but specifically bind to bacterial nucleic acids (DNA and RNA) or the peptidoglycan precursor lipid II, respectively [10, 12, 13].

Human granulysin as well as porcine and bovine NK-lysin are cationic AMPs [14–16]. NK-lysin and granulysin are found in the cytolytic granules of cytotoxic T-lymphocytes and NK cells that exhibit broad-spectrum antimicrobial activity against a variety of pathogens including Gram-positive and Gram-negative bacteria, fungi, viruses, and parasites [6, 15–18]. NK-lysin and granulysin are structurally similar and related to the saposin-like protein (SAPLIP) family of lipid binding proteins [15, 18, 19]. Potent antimicrobial activity of bovine NK-lysin peptides are associated with functional region helices 2 and 3 [16, 17, 20]. Although humans and swine have only a single granulysin or NK-lysin gene, the cattle genome has four functional NK-lysin genes, *NK1*, *NK2A*, *NK2B* and *NK2C* [17]. Gene expression analysis in bronchial lymph node and lung tissue samples of cattle challenged with bovine respiratory disease causing pathogens revealed that overall elevated expression of *NK2A* and *NK2C* as compared to healthy control cattle [17, 20].

Bovine respiratory disease complex (BRDC) or shipping fever, is the most common infectious disease affecting calves resulting in significant economic losses to the North American beef and dairy cattle industry [21, 22]. The etiology of BRDC is complicated by involvement of environmental stress factors, host factors, and various viral and bacterial infectious agents [23]. In conjunction with stress factors and active viral infections, calves are predispose to infection caused by commensal bacteria pathogens in the upper respiratory tract such as *Mannheimia haemolytica*, *Pasteurella multocida*, *Histophilus somni*, and *M*. *bovis* result in the BRDC development [21, 23]. *M*. *bovis* has been identified as a major contributor to respiratory disease and arthritis in feedlot cattle and dairy calves [24]. In addition to pneumonia, mastitis and arthritis, *M*. *bovis* is also known to cause otitis media, conjunctivitis, meningitis, and reproductive disorders [24, 25]. It has recently been recognized as a highly virulent pathogen in North American bison in which it has been reported to cause pneumonia, polyarthritis, necrotic pharyngitis, mastitis, dystocia and abortion [26, 27].

We and others have previously reported the antimicrobial activity of bovine NK-lysin peptides against several BRDC-causing bacterial pathogens [20, 28]. Although *M*. *haemolytica*, *P*. *multocida* and *H*. *somni* are closely related to each other since they are members of the family *Pasteurellaceae*, yet their sensitivity to NK lysin peptide mediated killing is very different: *H*. *somni* is the most sensitive and *P*. *multocida* is the least sensitive to NK lysin peptides[20, 28]. Although various studies have concluded that bovine NK-lysin-derived peptides cause disruption of the bacterial outer cell wall and inner cell membranes as well as leakage of intracellular contents, there have been only a few studies related to the sensitivity of *Mycoplasma* species to AMPs [29, 30]. To the best of our knowledge, there are no studies reporting on the susceptibility of *M*. *bovis* to bovine NK-lysin. Therefore, to fill this knowledge gap, the antimicrobial activity of bovine NK-lysin-derived peptides on bovine *M*. *bovis* isolates was evaluated. Furthermore, different assays were performed to elucidate the underlying mechanism of anti-*M*. *bovis* activity of bovine NK-lysin-derived peptides.

## Materials and methods

### Bovine NK-lysin-derived peptide synthesis

Linear peptides corresponding to the functional region helices 2 and 3 of bovine NK-lysins

NK1 [VIIHVTSKVCSKMGLWSILCNQMMKKYLNR, net charge = +5.0],

NK2A [TVIEVASKMCSKMRLLKGLCKSITKRFLRR, net charge = +7.9],

NK2B [TVIEAASKVCGKMGPLKGLCKSITKRFLRR, net charge = +6.9] and

NK2C [TVIEEASKVCSKMRLLKGLCKSIMKKFLRT, net charge = +5.9]) were synthesized by Peptide 2.0 Inc (Chantilly, VA) and supplied as trifluoroacetate salt with over 95% purity [16, 17, 28]. Lyophilized peptides were dissolved in Dulbecco’s phosphate-buffered saline (PBS, pH 7.1) without calcium or magnesium, aliquoted, and stored at -20°C until used.

### Circular dichroism (CD) assay

Each NK-lysin peptide (20 μM) was diluted in 10 mM potassium phosphate buffer (pH 7.4) either with liposome (1 mM) or without liposome and placed in a 1 mm path-length quartz cuvette (final volume = 300 μl). The negatively charged liposome containing phospholipids (35% POPE, 50% POPG and 15% cardiolipin) in potassium phosphate buffer solution (10 mM, pH 7.4) was purchased from Avanti Polar Lipids (Alabaster, AL). The secondary structures of NK-lysin peptides were then evaluated using a Jasco J-815 CD spectrophotometer (Jasco, Easton, MA). Measurements were taken every 0.1 nm from 250 to 190 nm with six accumulations at room temperature using automated baseline correction. Raw data in millidegrees was converted to molar ellipticity.

### Hemolytic activity assay

The hemolytic activity of NK-lysin peptides was studied using bovine red blood cells (RBCs) as described previously [31]. Briefly, 10 ml of cattle blood samples were collected into syringes containing acid citrate dextrose anticoagulant and transferred to 50 ml tubes. Blood samples were centrifuged (1000 × *g* for 10 min) and plasma and buffy coat were removed. Resulting RBC pellets were washed twice with PBS and resuspended in PBS to obtain 2.5% hematocrit. One hundred microliter aliquots of RBC suspensions were placed into a 96-well plate and mixed gently with 20 μl of each of the diluted NK-lysin peptides (5 and 30 μM final concentration, L_Exp_). Samples were incubated at 37°C in a humidified atmosphere for 60 min. The plate was centrifuged (1000 × *g* for 5 min) and 100 μl of each supernatant was transferred to a new 96-well plate and release of hemoglobin (from lysed RBCs) was monitored using a microplate reader by measuring the absorbance at 405 nm and 570 nm wavelengths. Triton X-100 (0.1% (v/v) final concentration, L_Tx100_) -treated RBC were used as a positive control (100% lysis) and PBS-treated RBCs (L_0_) were used as a negative control. The percentage of hemolysis was calculated by using the following formula (L_Exp_-L_0_)/L_Tx100_-L_0_) × 100 as described previously [31]. Means and standard error of mean were calculated from two independent experiments.

### Bacterial isolates and culture conditions

Bovine *M*. *bovis* isolate 428E [32] and bison isolate NADC1 [33] were maintained as freezer stocks (-80°C) in Difco™ PPLO broth without crystal violet (Becton, Dickinson and Company, Sparks, MD), supplemented with 10 g/l Bacto™ yeast extract (Becton, Dickinson and Company), and 20% (v/v) heat-inactivated horse serum (ThermoFisher, Waltham, MA). Working broth cultures, 3 ml each, were inoculated from freezer stocks and incubated overnight (~20 hrs) at 37°C in a humidified atmosphere of 5% CO_2_.

### Antimicrobial killing assay

An antimicrobial killing assay was performed as described previously, but with minor modifications [16, 20, 28]. Briefly, overnight cultures of *M*. *bovis* isolates grown in PPLO broth were diluted (1:100) in PBS to obtain ~1 × 10^6^ colony forming units per milliliter (CFU/ml). One hundred microliters of each *M*. *bovis* suspension (~1 × 10^5^ CFUs) were placed in a non-tissue culture treated, flat-bottom, 96-well plate (Becton, Dickinson and Company) and gently mixed with 20 μl of PBS (for negative controls) or the indicated NK-lysin peptide, diluted to a final concentration of 2, 5, 10, 20, or 30 μM. The plate was covered with a lid and incubated at 37°C in a humidified atmosphere of 5% CO_2_ for 60 min. Bacterial-peptide samples were serially diluted in PBS and 5 μl of each dilution was plated on Difco™ PPLO agar plates, in triplicate. Plates were incubated at 37°C in a humidified atmosphere of 5% CO_2_ for 3 days, until colonies were easily visualized under 25× magnification. The number of CFU in 5 μl was enumerated for all dilutions yielding visually distinguishable colonies and used to calculate the average number of CFU/ml in the original sample or control. The percentage of viable cells remaining after treatment with NK-lysin peptides was calculated by dividing the average number of CFU/ml in each sample by the average number of CFU/ml in the corresponding negative control, × 100. Final percentages for each peptide are based on three independent replicates of the killing assay.

### Membrane depolarization assay using DiSC_3_(5) fluorescent dye

To determine whether bovine NK-lysin peptides have the ability to disrupt electrochemical potential across the *M*. *bovis* membrane, a cytoplasmic membrane depolarization assay was performed using a membrane potential-sensitive cyanine dye 3,3'-dipropylthiadicarbocyanine iodide (DiSC_3_(5); AnaSpec, Fremont, CA) as described previously, but with some modifications [34–36]. Briefly, *M*. *bovis* was harvested from overnight cultures by centrifugation (10,000 × *g* for 10 min) and washed once with 5 mM HEPES-20 mM glucose buffer, pH 7.4. *Mycoplasma bovis* (~1 × 10^8^ CFUs/ml) was resuspended in the same buffer, DiSC_3_(5) (0.4 μM final concentration) was added, gently mixed by pipetting, and 100 μl aliquots (~1 × 10^7^ CFUs) were transferred into a 96-well opaque plate and incubated at 37˚C in a humidified atmosphere of 5% CO_2_ to obtain stable reduction of DiSC_3_(5) fluorescence (~30 min). To equilibrate cytoplasmic and external K^+^ concentrations, 100 μl of 200 mM KCl, pH 7.4 was added into each well and incubated for another 10 min. NK-lysin peptides (5 and 20 μM final concentrations) were added and changes in fluorescence were immediately measured using a fluorescent microplate reader with excitation and emission wavelengths at 622 nm and 670 nm, respectively. Triton X-100 (0.1% (v/v) final concentration) treated *M*. *bovis* was used as a positive control, while ethanol treated (killed) and untreated *M*. *bovis* (in PBS) were used as negative controls.

### Bacterial viability staining

To distinguish dead bacteria from live bacteria following incubation with NK-lysin peptides, *M*. *bovis* was stained using the LIVE/DEAD *Bac*Light bacterial viability kit as described by the manufacturer (ThermoFisher Scientific, Carlsbad, CA), but with some modifications. Briefly, ~1 × 10^7^ cfu of *M*. *bovis* in 100 μl of PBS was placed in a 96-well plate and 5 μM and 20 μM final concentrations of each of the NK-lysin peptides was added and samples were incubated at 37°C in a humidified atmosphere of 5% CO_2_ for 30 min. Fifty microliters of PI and 50 μl of Syto 9 (approximate final concentrations of Syto 9 and PI were 6 μM and 30 μM, respectively) were added to each well and incubated at 37°C for additional 15 min. The viability of *M*. *bovis* was then determined by confocal laser-scanning microscopy and flow cytometry (green (live) versus red (dead) bacteria). Untreated and ethanol killed *M*. *bovis* were used as live and dead controls, respectively.

### Flow cytometry

Two-color flow cytometric analyses was performed using a BD LSRII flow cytometer (BD Biosciences). *M*. *bovis* were visualized in forward and side light scatter and electronic gates were set to contain bacteria at the single cell level. Single (Syto 9 or PI) and dual fluorescence dye labeled bacteria, in addition to use of compensation beads, were included to optimize acquisition gates and compensation for each fluorochrome. Both Syto 9 and PI were excited at 488 nm laser beam and the emission signals were detected using a 530/30 nm and 575/25 nm long-pass filters, respectively. Approximately, 10,000 events were collected for data analysis and relative changes in live and dead bacteria were determined using FlowJo software (FlowJo LLC, Ashland, OR).

### Confocal laser-scanning microscopy

Approximately 150 μl of bacterial suspensions, previously incubated with NK-lysin or PBS along with PI (but no Syto 9), were adhered to glass slides using a Shandon cytospin 2. Coverslips were mounted using ProLong^®^ Gold antifade mount medium with DAPI (4`, 6`-diamidino-2-phenylindole, ThermoFisher). Bacteria were visualized using a Nikon A1R+ laser scanning confocal microscope (Nikon Instruments, Melville, NY). DAPI and PI were excited at 405 nm and 561 nm single state laser diode and emission signals 425–475 nm (blue) and 570–620 nm (red) were recorded, respectively. Images were collected using Nikon’s NIS-Elements Advanced Research software. Calibration was created for both fluorescent dyes using the software and sequentially collected frames of individual channels were merged and saved as TIFF files. Images were obtained with plan Apo 60× objective lens (oil) at numerical aperture 1.4. Final figures were prepared using Adobe Photoshop Elements 11.

### Propidium iodide uptake assay

The kinetics of NK-lysin-induced damage to the *M*. *bovis* cell membrane were determined using the propidium iodide (PI) uptake assay as described previously [28, 29]. Briefly, *M*. *bovis* (~1 × 10^8^ CFUs/ml) was harvested from overnight cultures by centrifugation (10,000 × *g* for 10 min) and washed once with PBS. Approximately 1 × 10^7^ CFUs of *M*. *bovis* in 100 μl PBS was placed in a 96-well opaque plate and mixed with 5 μl of PI (3 μM final concentration). Samples were then incubated at 37°C in a humidified atmosphere of 5% CO_2_ for 5 min followed by the addition of NK-lysin peptides (5, and 20 μM final concentrations). At predetermined time points (1, 3, 10, 15, and 30 min), PI fluorescent intensity of each sample was measured using a fluorescent microplate reader with excitation and emission wavelengths at 530 nm and 620 nm, respectively. Triton X-100 (1.0% (v/v) final concentration) and PBS were used as positive and negative controls, respectively. Means and standard error of mean were calculated from two independent experiments.

### Transmission electron microscopy

Approximately, 1 × 10^8^ CFUs of *M*. *bovis* in 100 μl of PBS was transferred to a non-tissue culture treated flat-bottom 96-well plate and incubated with 30 μM of NK-lysin peptides or PBS at 37°C in a humidified atmosphere of 5% CO_2_ for 60 min. The bacterial suspension was mixed with an equal volume of 3% glutaraldehyde in 0.1 M cacodylate buffer, pH 7.4 and processed for transmission electron microscopy as described previously [17, 28]. Briefly, after fixation, bacterial pellets were rinsed in cacodylate buffer, post-fixed in 1% osmium tetroxide, dehydrated in alcohols, and embedded in epoxy resin. Ultrathin sections of the bacterial pellets were cut and stained with uranyl acetate and lead citrate. Sections were examined with a FEI Tecnai G2 Biotwin (FEI Co., Hillboro, OR) transmission electron microscope and images were taken with Advanced Microscopy Technologies (AMT Inc., Danvers, MN) imaging camera.

### Statistical analysis

The mean number of viable bacteria and standard errors of means were determined using Microsoft Excel software. Student’s *t*-test and analysis of variance (ANOVA) were used to compare differences between treatments. The term significant indicates *P* value of less than 0.05.

## Results

### Structural changes of bovine NK-lysin peptides in liposomes

**Fig 1:**
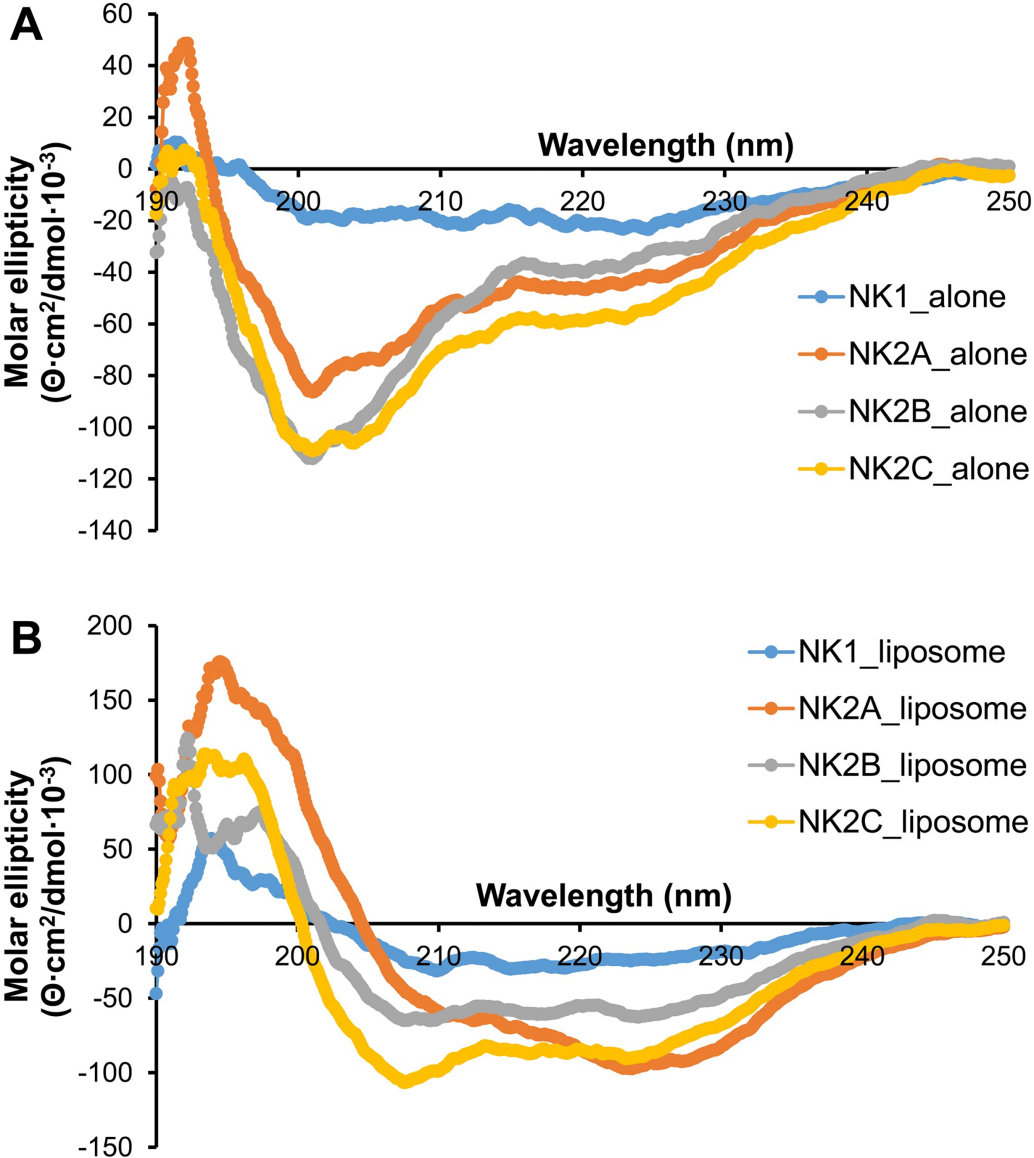
Circular dichroism (CD) spectral analysis of bovine NK-lysin peptides. CD spectra of all four bovine NK-lysin peptides (20 μM final concentrations) were determined in the absence of (A) or presence (B) of liposomes (1 mM final concentration) in 10 mM potassium phosphate buffer solution. CD spectra were measured at room temperature using Jasco J-815 CD spectrometer and measurements were taken every 0.1nm from 250 to 190nm. Average spectra with six accumulations after baseline correction for each of the peptides are shown.

Circular dichroism (CD) spectroscopy has been widely used to estimate the conformation or the secondary structures of peptides and proteins in solution [37]. We have also taken the advantage of this technique to determine the secondary structures of bovine NK-lysin-derived peptides. All four peptides when resuspended in potassium phosphate buffer alone (without liposomes) showed CD spectra consistent with random coil structures (minima near 200 nm; Fig 1A). All four peptides resuspended in 1 mM liposomes showed characteristic CD spectral changes indicative of α-helical structures (maxima near 192 nm a double minima near 208 nm and 222 nm; Fig 1B) suggesting conformational changes of NK-lysin peptides upon binding with lipid membranes. However, compared to NK2 peptides, NK-1 peptide showed a lower degree of α helicity (Fig 1B).

### Hemolytic activity of bovine NK-lysin peptides

**Fig 2:**
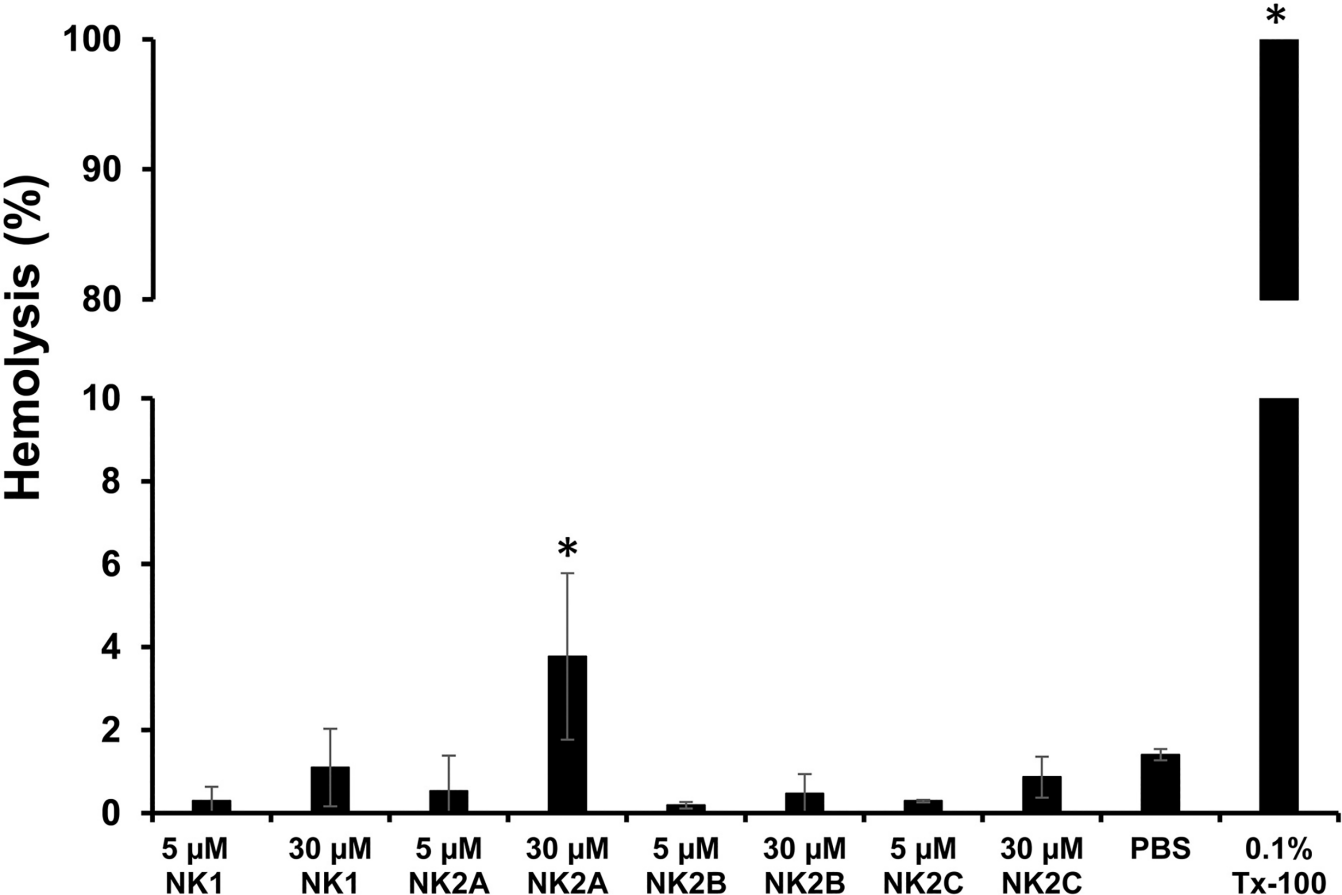
Hemolytic activities of bovine NK-lysin peptides on cattle erythrocytes. The cattle RBCs were resuspended in PBS (2.5% final hematocrit) and incubated with 5 μM and 30 μM bovine NK-lysin peptides at 37°C in a humidified atmosphere for 60 min. The release of hemoglobin was measured spectrophotometrically at 405 nm and 570 nm wavelengths. RBCs incubated with PBS or 0.1% (v/v) Triton X-100 served as negative and positive controls, respectively. Means and SEM were calculated from two independent experiments. (* = *P*<0.007).

In order to identify whether bovine NK-lysin peptides have any hemolytic properties, hemoglobin release assay was performed using cattle RBCs. As expected, control cattle RBCs incubated with PBS had only a minimal hemolysis (spontaneous) whereas, incubation of RBCs with Triton X-100 (0.1% (v/v)) resulted in complete hemolysis. All four NK-lysin peptides, no matter the concentration (5–30 μM), had less than 6% hemolytic activity (Fig 2).

### Antimicrobial activity of bovine NK-lysin peptides on *M*. *bovis*

**Fig 3:**
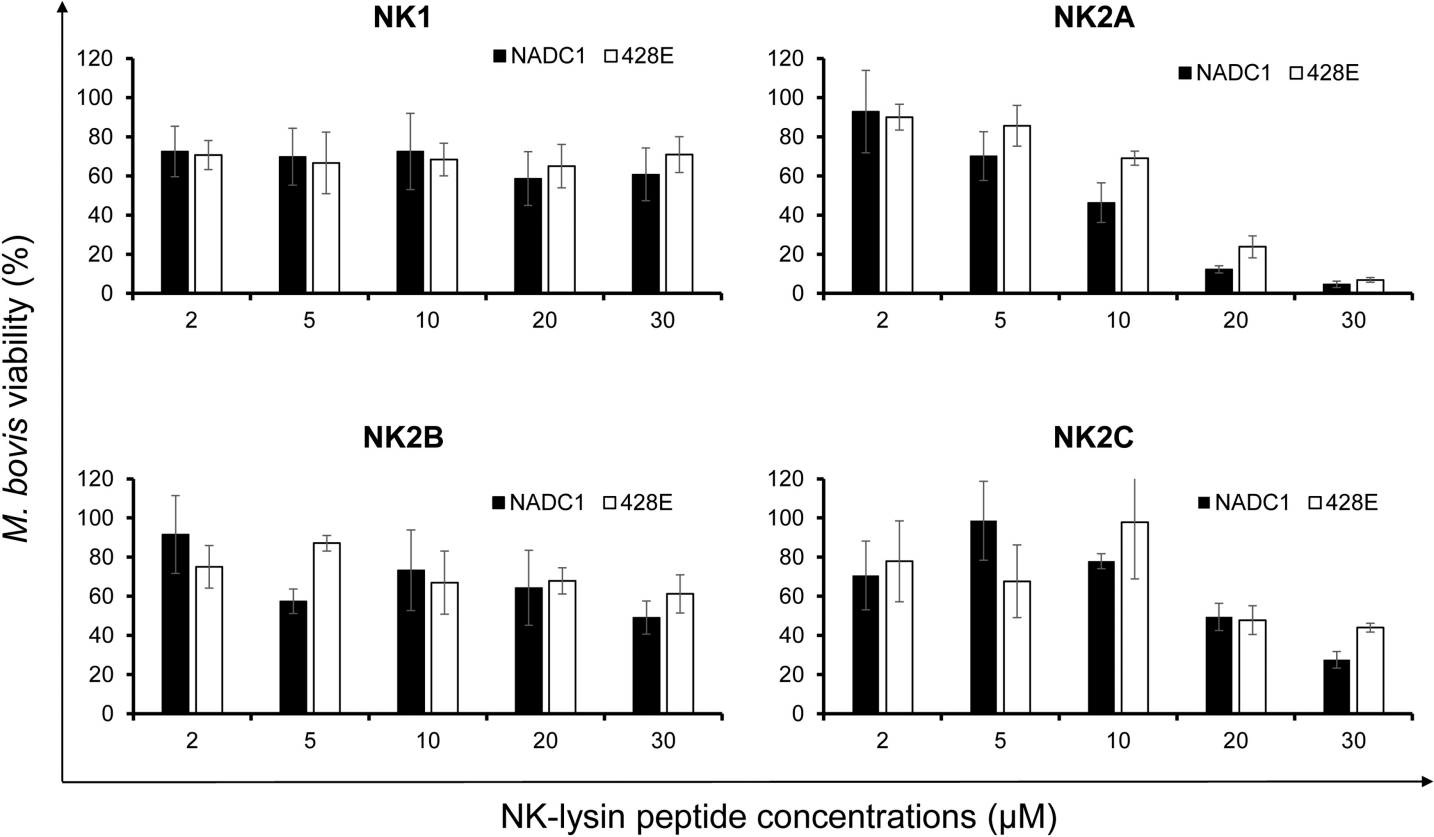
Antimicrobial activity of bovine NK-lysin peptides on *M*. *bovis*. A bovine (428E) or bison (NADC1) isolate of *M*. *bovis* was incubated with the indicated concentrations of bovine NK-lysin peptides at 37°C in a humidified atmosphere of 5% CO_2_ for 60 min. The percentage of viable cells remaining was calculated in comparison to the same isolate incubated under identical conditions with only PBS. Results shown are the means and SEM of three independent experiments.

The antimicrobial activity of bovine NK-lysin-derived peptides against cattle BRDC causing bacterial pathogens such as *M*. *haemolytica*, *P*. *multocida* [20] and *H*. *somni* [28] have been previously reported. It was of our interest to assess whether bovine NK-lysin peptides demonstrate any antimicrobial activity against *M*. *bovis*, another major contributor to the BRDC. We used 30-mer peptides corresponding to the functional region of bovine NK-lysin helices 2 and 3 of NK1, NK2A, NK2B and NK2C for the killing assays [16, 17, 28]. Overall, *M*. *bovis* isolate NADC1 was slightly more sensitive to bovine NK-lysin peptides mediated killing compared to *M*. *bovis* isolate 428E (Fig 3). Among four peptides tested, NK1 and NK2B were the least effective against both *M*. *bovis* isolates even at higher peptide concentrations (20–30 μM). Although both NK2A and NK2C peptides showed higher antimicrobial activity against both *M*. *bovis* isolates, NK2A showed the highest antimicrobial activity (Fig 3).

### Membrane depolarization activity of NK-lysin peptides on *M*. *bovis*

**Fig 4:**
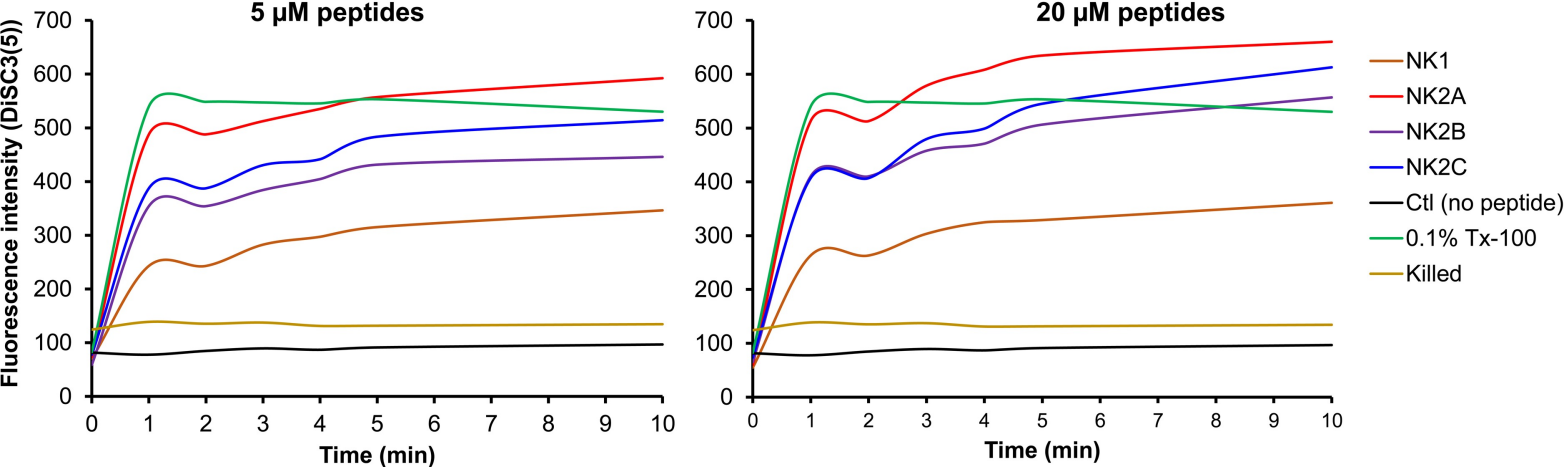
Plasma membrane depolarization activity of bovine NK-lysin peptides on *M*. *bovis*. *M*. *bovis* was incubated with membrane-potential sensitive cyanine dye DiSC_3_(5) (0.4 μM final concentration) at 37°C for 30 min followed by addition of KCl (100 mM final concentration). Bovine NK-lysin peptides (5 and 20 μM final concentrations) were added and changes in fluorescence were immediately measured using a fluorescent microplate reader with excitation and emission at 622 nm and 670 nm wavelengths, respectively. Triton X-100 (0.1% (v/v) final concentration) was used as a positive control, while ethanol treated (killed) and untreated *M*. *bovis* (in PBS) were used as negative controls. Results of one representative experiment out of three are shown.

It has been previously revealed that cyanine dyes lose fluorescence intensity in polarized membranes and become highly fluorescent once the membrane is depolarized [35]. Since the bacterial membrane depolarization properties of bovine NK-lysins have not been reported previously, the ability of bovine NK-lysins to breach the *M*. *bovis* plasma membrane barrier leading to depolarization was studied in this study. The intensity of the fluorescence signal started to decrease as DiSC_3_(5) dye was incorporated into the polarized cytoplasmic membrane and cytoplasm of *M*. *bovis* and steady baseline of fluorescence intensity was achieved at 30 min incubation. Fluorescence intensity increased immediately after the addition of all four bovine NK-lysin peptides at both lower (5 μM) and higher (20 μM) concentrations indicating *M*. *bovis* plasma membrane depolarization (Fig 4). Maximum depolarization of *M*. *bovis* cytoplasmic membrane was reached within approximately 5 min with all four peptides (Fig 4). However compared to three NK2 peptides, NK1 peptide showed the lowest fluorescent signal (Fig 4). As anticipated, killed *M*. *bovis* (ethanol treated) and *M*. *bovis* incubated with PBS (negative control) did not show membrane depolarization, whereas incubation of *M*. *bovis* with 0.1% (v/v) Triton X-100 resulted in an immediate membrane depolarization (Fig 4).

### Bovine NK-lysin peptides induce damage to *M*. *bovis* plasma membrane

**Fig 5:**
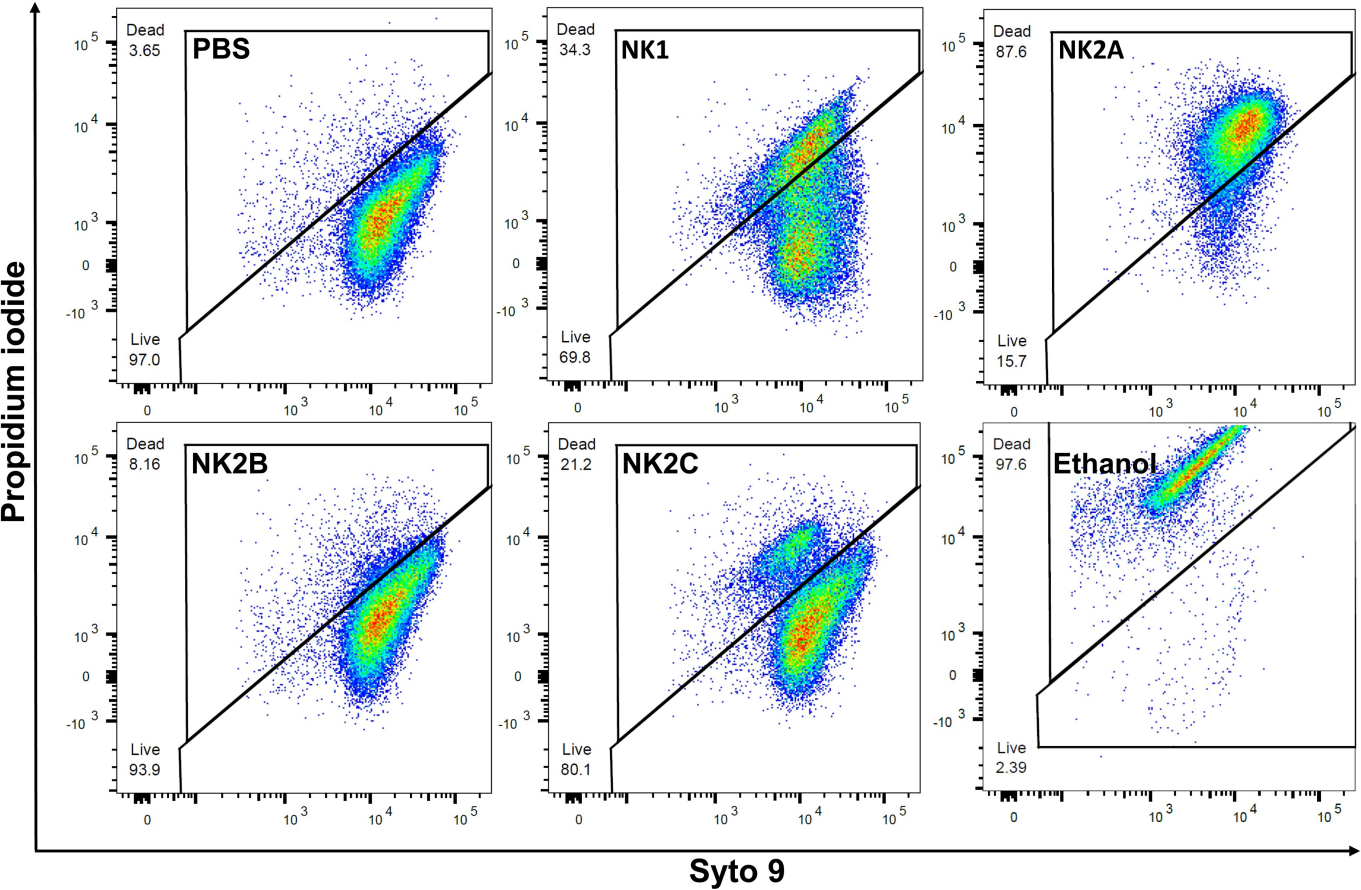
Flow cytometric analysis of *M*. *bovis* treated with bovine NK-lysin peptides. Live and dead *M*. *bovis* in PBS, NK1, NK2A, NK2B, NK2C, and ethanol treated samples. *M*. *bovis* was incubated with 20 μM of NK-lysin peptides at 37°C for 30 min followed by staining with Syto 9 and PI. X axis indicates live bacteria (Syto 9) with intact membranes and Y axis indicates dead bacteria (PI) with damaged membranes. Results of one representative experiment out of three are shown.

**Fig 6:**
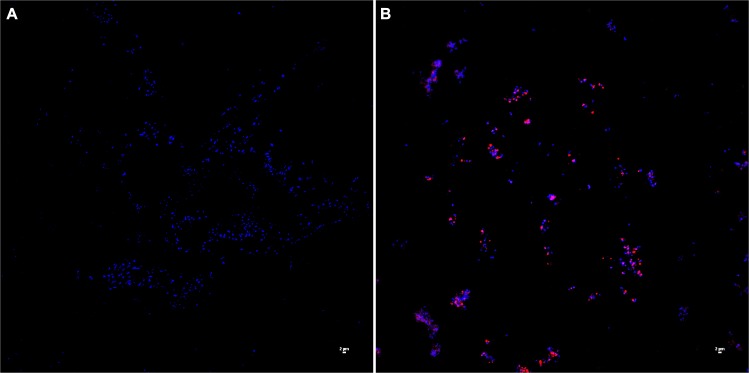
Confocal laser-scanning microscopic analysis of *M*. *bovis* treated with bovine NK-lysin peptides. Live and dead *M*. *bovis* in PBS (A), and NK2A (B) samples. *M*. *bovis* was incubated with 20 μM NK2A at 37°C for 30 min, and then stained with PI and DAPI. Dead bacteria with damaged membranes (red) were stained with PI and DAPI while live bacteria were stained only with DAPI. Results of one representative experiment out of three are shown.

*M*. *bovis* was sensitive to NK-lysin-derived peptides as evidenced by a reduction in bacterial viability in killing assays (Fig 3); however, it was not clear whether the NK-lysin peptides can damage the structural integrity of the *M*. *bovis* plasma membrane. Therefore, we performed Live/Dead bacterial viability staining with Syto 9 and PI after the incubation of *M*. *bovis* with NK-lysin peptides. Syto 9 is a cell permeant fluorescent dye (green) which can efficiently bind with cellular DNA with intact cell membranes. PI is a cell non-permeant fluorescent dye (red) and only stains DNA in the cells with compromised cell membranes. As assessed by flow cytometry, the majority of bacteria in the control sample stained with Syto 9 and ethanol-killed bacteria stained with PI (Fig 5). These findings suggested that unlike ethanol-killed bacteria, the plasma membranes of live *M*. *bovis* were intact. Similar to the killing assay results, the highest number of PI-positive (or dead) *M*. *bovis* was found in NK2A treated sample while the lowest PI positive cells were found in NK2B-treated sample (Fig 5). Concurrently, similarly incubated *M*. *bovis* samples were also analyzed by confocal microscopy. Again, as expected, bacteria in the control sample were largely negative for PI staining (Fig 6A), whereas the majority of *M*. *bovis* incubated with NK2A peptide were appeared to be positive for PI staining (Fig 6B).

**Fig 7:**
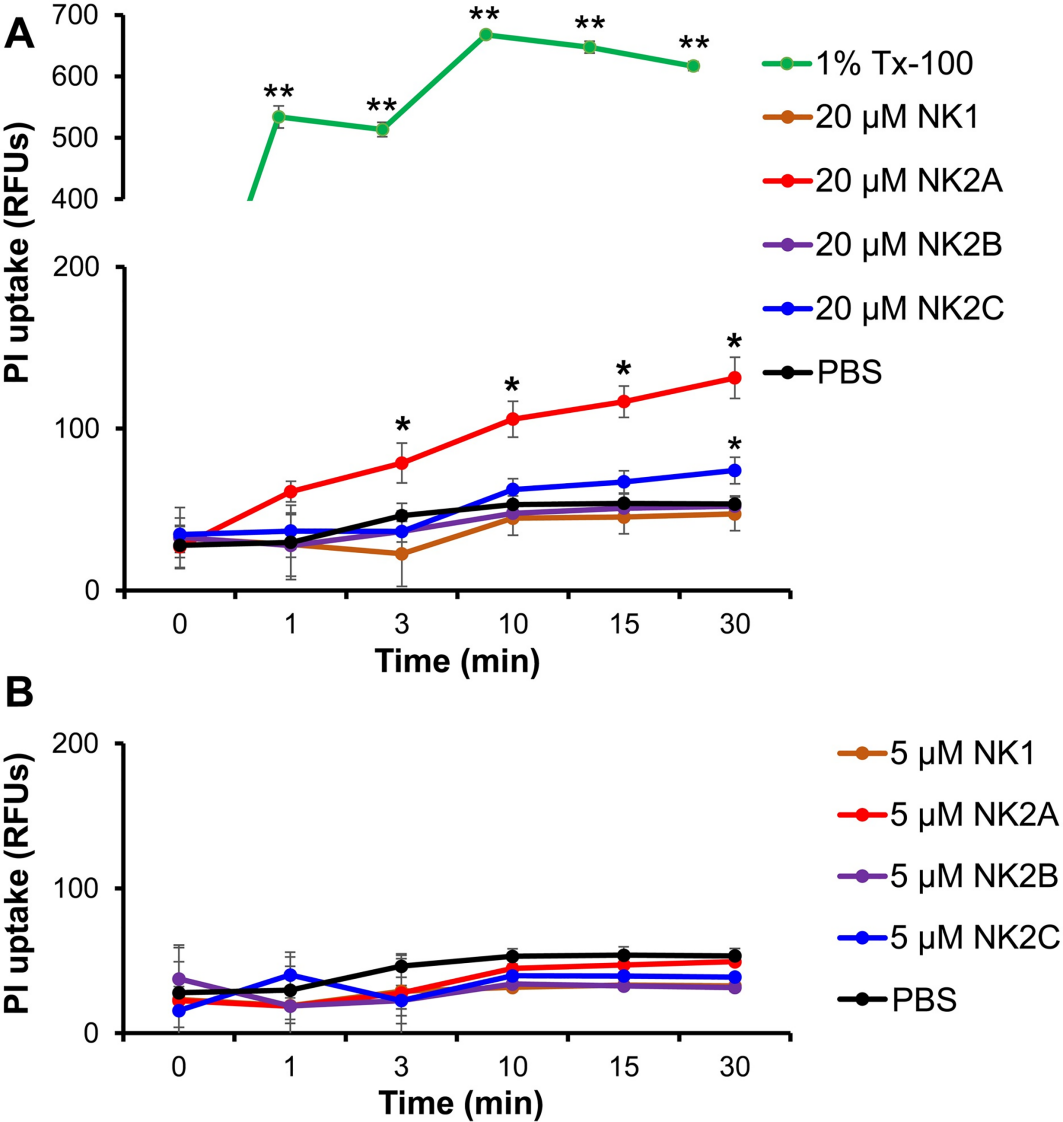
PI uptake of *M*. *bovis* following incubation with bovine NK-lysin peptides. *M*. *bovis* was incubated with PI (3 μM final concentration) for 5 min followed by addition of 20 μM (A) and 5 μM (B) final concentrations of NK-lysin peptides and PI fluorescence was immediately measured using a fluorescent microplate reader with excitation and emission at 530 nm and 620 nm wavelengths, respectively. Triton X-100 (1% v/v) was used as a positive control and PBS was used as a negative control. Results shown are the means and SEM of three independent experiments. Symbols *, and ** indicate significant differences in PI uptake compared to the PBS-treated control (** = *P*<0.001; * = *P*<0.05).

In order to further understand the kinetics of NK-lysin-induced *M*. *bovis* plasma membrane damage as well as killing activity, a PI uptake assay was performed in a time- and a dose-dependent manner following incubation of *M*. *bovis* with two different concentrations of peptides (5 μM and 20 μM). As anticipated, higher PI uptake was observed with *M*. *bovis* treated with Trion X-100 (1% v/v) while very low PI uptake was detected with control (no peptide) *M*. *bovis* samples (Fig 7A and 7B). As early as 3 min post-incubation, the level of PI uptake observed in *M*. *bovis* samples treated with 20 μM NK2A was significantly greater than the level of PI uptake in untreated cells and PI uptake continuously increased during the 30 min incubation period (Fig 7A). Lower, but continuously increasing PI uptake for *M*. *bovis* treated with 20 μM NK2C was also observed (Fig 7A). PI uptake in NK2A- and NK2C-treated *M*. *bovis* samples was lower than *M*. *bovis* treated with Triton X-100. No significant PI uptake was observed in the cells treated with 20 μM NK1 or NK2B (Fig 7A) nor with any peptide when tested at 5 μM (Fig 7B). Taken together, flow cytometric, confocal microscopic, and PI uptake findings strongly suggest that NK-lysin peptides can induce membrane permeabilization leading to pore formation in *M*. *bovis* plasma membranes.

**Fig 8:**
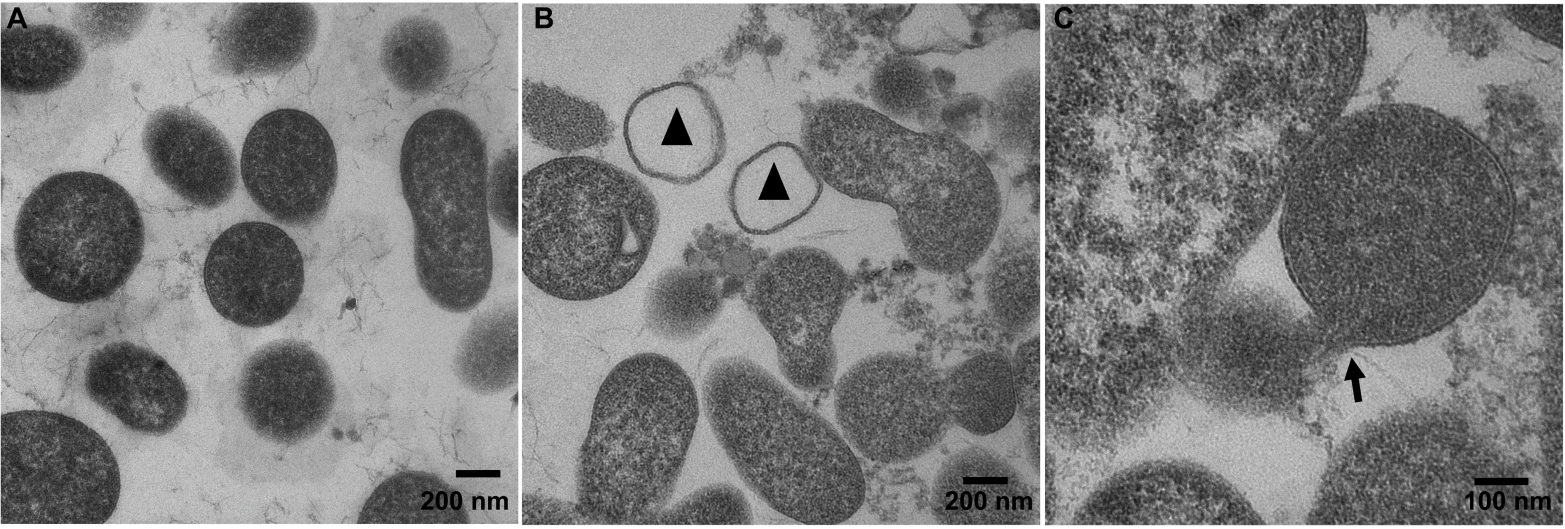
NK-lysin-induced damage to *M*. *bovis* plasma membrane. *Mycoplasma bovis* was incubated with PBS (A: control), 30 μM NK2A (B) or 30 μM NK2C (C) at 37°C for 60 min and processed for TEM. *M*. *bovis* incubated with NK-lysin peptides show damaged membranes as evidenced by ghost cells (triangles in panel B) and ruptured membranes (arrow in panel C). Control *M*. *bovis* (panel A) did not have any damaged membranes or ghost cells.

In order to visualize potential NK-lysin-mediated ultrastructural changes to the *M*. *bovis* plasma membrane, control and NK-lysin-treated *M*. *bovis* samples were prepared for transmission electron microscopic evaluation. Plasma membranes of *M*. *bovis* controls incubated in PBS appeared undamaged and their cytosols were filled with electron-dense material indicating these bacteria were alive and structurally intact (Fig 8A). We did not see a major difference in cytosolic electron densities between PBS- and NK-lysin treated *M*. *bovis* samples (Fig 8). However, cells with damaged plasma membranes, as evidenced by formation of pores, and leakage of intracellular contents were observed in NK2A- (Fig 8B) and NK2C-treated (Fig 8C) samples. Furthermore, a small number of ‘ghost cells’ in which there was a complete absence of intracellular contents (Fig 8B) was also observed in both NK2A- and NK2C-treated samples; such changes were not apparent in PBS-treated control cells.

## Discussion

*M*. *bovis* is a member of the family *Mycoplasmataceae* in the class *Mollicutes*. In general, members of the genus *Mycoplasma* are the smallest and simplest self-replicating bacteria. Unlike other bacteria that are associated with BRDC such as *M*. *haemolytica*, *P*. *multocida*, and *H*. *somni*, *M*. *bovis* does not have a cell wall around its plasma membrane. Although the US *M*. *bovis* isolates showed higher sensitivity to various antibiotics including erythromycin [38], *M*. *bovis* isolates from the Netherlands exhibit reduced sensitivity to antibiotics including macrolides and fluoroquinolones [39]. Since only a handful of antibiotics are available or approved for use in cattle, it is important to identify new antibiotics or alternatives to antibiotics reagents to effectively treat cattle or bison infected with *M*. *bovis*. In this context, we have measured the susceptibility of *M*. *bovis* cattle and bison isolates to bovine NK-lysin-derived peptides.

We and others have previously reported the differential sensitivity of members of the family *Pasteurellaceae* such as *M*. *haemolytica*, *P*. *multocida*, and *H*. *somni* to antimicrobial activity of bovine NK-lysin peptides [20, 28]. Although *M*. *haemolytica* was highly sensitive to NK2A and NK2C peptides, *P*. *multocida* was moderately sensitive to NK1 and NK2A peptides [20]. Compared to *M*. *haemolytica* and *P*. *multocida* isolates, *H*. *somni* isolates showed higher sensitivity to all four NK-lysin peptides [28]. Although one could expect higher sensitivity of bovine *M*. *bovis* isolates to NK-lysin peptides due to the lack of a cell wall, this was not the case. The 2 *M*. *bovis* isolates examined in this study were sensitive mainly to NK2A and NK2C peptides, but at relatively high concentrations (20–30 μM). In order to determine whether bovine NK-lysin-derived peptides have any cytotoxic effects on mammalian cells, hemolytic activity assay was performed with cattle RBCs. All four peptides showed lower hemolytic activity at 5 μM. However, approximately two fold increased in hemolytic activity was observed with all four peptides at 30 μM final concentration but the hemolytic activity was less than 6%. Therefore, due to the lower hemolytic activity on cattle RBCs and higher bactericidal activity against various pathogens including *M*. *bovis*, NK-lysin peptides appeared to have selective activity against microbial pathogens.

NK2A, NK2B and NK2C amino acids sequences are highly similar to each other as compared to NK1. Although all four synthetic NK-lysin peptides (corresponding to the functional region helices 2 and 3) showed similar hydrophobicity (40–43%), basic residues (20–30%), and net positive charges (5.0–7.9), NK2 peptides showed a higher degree of α-helicity compared to NK1 peptide upon binding with liposomes. However, NK1 peptide was highly effective against *E*. *coli* and *S*. *aureus* [17]. Despite having a higher degree of α-helicity in all three NK2 peptides, NK2B peptide was not very effective against *M*. *bovis* (this study) and *M*. *haemolytica* isolates [20], but all three NK2 peptides (including NK2B) were highly effective against *H*. *somni* isolates [28]. Therefore, the degree of α-helicity of NK-lysin peptides appears not to be directly associated with their antimicrobial activity.

The prokaryotic plasma membrane typically lacks sterols. *Mycoplasma* species however are known to incorporate sterols such as cholesterol (35–50% of total lipids in the membrane) into their plasma membrane either from serum in the growth medium or from the host to maintain membrane rigidity [40]. The exact lipid composition of the *M*. *bovis* plasma membrane is unknown at this moment. The plasma membranes of *M*. *pulmonis* and *M*. *hyorhinis* are known to contain five major types of lipids such as neutral lipids (unesterified cholesterol), cardiolipin, phosphatidylglycerol, phosphatidylcholine, and sphingomyelin [29, 41]. Given the similarities in growth requirements of various *Mycoplasma* species, we could expect similar phospholipid composition in *M*. *bovis* plasma membrane as well. Changes to the composition of the plasma membrane lipid bilayer have been shown to influence the antimicrobial activity of certain AMPs such as ceragenins (CSA-13) and cathelicidin LL-37 [29, 42]. A recent review article revealed that cholesterol is believed to play a bigger role (compare to other types of lipids in bacterial membranes) sensitivity to AMPs [43]. Therefore, given the similarities in amino acid residues and secondary (helical) structures among four bovine NK-lysin peptides, observed differences in sensitivity of *M*. *bovis* and other bacterial pathogens to these peptides are likely to be associated with differences in the lipid composition of the bacterial plasma membrane. Indeed, findings from several studies revealed that high concentrations of AMPs such as cathelicidin or cathelin-related peptides are required to kill *M*. *pulmonis* and *M*. *pneumoniae* [29, 44] as compared to several Gram-positive (ex: *Listeria monocytogenes*, *Bacillus megaterium*, and Group A-C *Streptococcus*) and -negative bacterial (ex: *Salmonella enterica* subsp. *enterica serovar* Minnesota, *Klebsiella pneumoniae*, and *Proteus vulgaris*) pathogens [45].

It has been previously reported that the existence of larger electrical potential across the bacterial cytoplasmic membrane and disruption of the membrane barrier allow flow of charge without necessarily allowing the passage of small molecules [46]. Dermcidin-derived peptides and ceragenins (CSA-8 and CSA-54) are known to kill bacteria simply by membrane depolarization and without the induction of membrane permeabilization or pore formation [46, 47]. We and others have previously shown that bovine NK-lysin-derived peptides can disrupt both outer- and inner-membranes of BRDC-causing bacterial pathogens [20, 28]. However, no studies have so far been reported whether NK-lysin-derived peptides can induce bacterial membrane depolarization. Membrane potential-sensitive cyanine dye such as DiSC_3_(5) has been widely used to determine the depolarization properties of various AMPs [34–36]. In this assay, release of plasma membrane bound DiSC_3_(5) into the medium indicates membrane depolarization. All four NK-lysin peptides at both higher (20 μM) as well as lower (5 μM) peptide concentrations were able to dissipate *M*. *bovis* membrane potential leading to membrane depolarization as identified in DiSC_3_(5) assay. However, unlike dermcidin-derived peptides and ceragenins, this effect appears not to be bactericidal. Only 20 μM NK2A and NK2C (but not NK1 or NK2B) resulted in significant PI uptake indicating damage to the plasma membrane presumably by pore formation. Indeed, NK2A- and NK2C-induced pore formation was of the *M*. *bovis* plasma membrane was clearly visible in electron micrographs. All four NK-lysin peptides are highly effective against various other bacterial pathogens and have showed similar secondary structures. The reason for lack of *M*. *bovis* killing by NK1 and NK2B is unknown.

Taken together, the findings in this study suggest that NK-lysin-induced *M*. *bovis* plasma membrane depolarization alone was insufficient for killing and similar to most other AMPs, NK-lysin-mediated disruption of *M*. *bovis* plasma membrane appears to be involved in the killing.

### Conclusions

In order to identify the mechanism of antimicrobial action of bovine NK-lysin-derived peptides on *M*. *bovis*, we used a bacterial killing assay, flow cytometry, confocal laser-scanning microscopy, and PI uptake along with membrane depolarization assays. Analyses of these assays revealed that NK-lysin peptides are bactericidal to *M*. *bovis* isolates. Although all four NK-lysin peptides were able to breach *M*. *bovis* plasma membrane permeability barrier leading to membrane depolarization, such depolarization alone was insufficient to kill *M*. *bovis*. Electron microscopic analyses confirmed cells with ruptured plasma membranes upon incubation of *M*. *bovis* with NK2A and NK2C peptides. Based on our results with 2 *M*. *bovis* isolates and those observed by others previously [20], we can conclude in *in vitro* models that bovine NK-lysin-derived peptides (NK2A and NK2C) are effective against most bacterial agents involved in BRDC [20, 28]. The fact that NK2C is expressed strongly in lung suggests that NK2C is most likely the NK-lysin of interest in future BRDC testing. Testing bovine NK-lysin-derived peptides in real world animal disease models will be required to determine in NK-lysins have a role in animal disease resolution.
